# Reading the lipid code: Cooperative domain functions in perilipin targeting to lipid droplets

**DOI:** 10.1016/j.jbc.2026.113619

**Published:** 2026-10-08

**Authors:** Jiri Stribny, Roger Schneiter

**Affiliations:** Department of Biology, University of Fribourg, Chemin du Musée 10, Fribourg, Switzerland

**Keywords:** amphipathic helices, 11-mer repeats, lipid packing defects, neutral lipids, surface tension, nascent LDs, metabolic dysfunction-associated steatotic liver disease (MASLD), apolipoprotein E (ApoE)

## Abstract

Perilipins (PLINs) are the most abundant proteins coating the phospholipid monolayer of lipid droplets (LDs), regulating their biogenesis, stability, growth, and lipolysis. Most mammalian isoforms (PLIN1–3, PLIN5) share a modular architecture characterized by an N-terminal PAT domain (perilipin-adipophilin-TIP47), a central 11-mer repeat region forming amphipathic helices (hydrophobic and hydrophilic faces on opposite sides of the helix), and a C-terminal 4-helix bundle; PLIN4 is a notable exception that relies mainly on expanded amphipathic helices. In this review, we synthesize recent structural, biophysical, and cell-biological advances and present a working framework in which these domains function cooperatively to read a local lipid code composed of diacylglycerol (DAG) enrichment, phospholipid composition, packing defects, and surface tension. The PAT domain preferentially engages DAG-rich endoplasmic reticulum (ER) subdomains to initiate recruitment and support LD budding. The 11-mer amphipathic helices, often weakly hydrophobic and highly repetitive, especially in PLIN4, recognize lipid packing defects and enable dynamic, tension-responsive binding, with polar residues supporting multimeric assembly and exceptional stability on LD surfaces. The C-terminal 4-helix bundle contains a conserved hydrophobic cleft that can accommodate phosphatidylethanolamine (PE); isolated domains prefer PE-rich interfaces, and PE limitation reduces cellular recruitment. Isoform-specific variations, including PLIN1’s integral membrane segment for ER insertion and high-affinity binding, contribute to hierarchical sorting and LD heterogeneity. Together, these cooperative domain functions integrate lipid cues to orchestrate LD assembly and remodeling, with implications for metabolic diseases such as metabolic dysfunction-associated steatotic liver disease, obesity, and lipodystrophies.

Lipid droplets (LDs) are dynamic, evolutionarily conserved organelles that serve as the primary intracellular storage sites for neutral lipids, primarily triacylglycerols (TAG) and steryl esters. Unlike bilayer-bound organelles, LDs possess a unique phospholipid monolayer that shields the hydrophobic core from the aqueous cytosol (1). This monolayer is functionalized by a specific set of proteins, among which the perilipins (PLINs) constitute the most abundant coat proteins (2). PLINs regulate LD biogenesis, stability, growth, and mobilization, thereby maintaining cellular lipid homeostasis (3). Dysregulation of PLIN function is associated with metabolic disorders, including metabolic dysfunction-associated steatotic liver disease (MASLD), obesity, lipodystrophies, and cancer 4, 5.

Mammals express five PLIN isoforms (PLIN1–PLIN5) with tissue-specific expression and functional specialization. Most isoforms share a conserved modular architecture consisting of an N-terminal PAT (perilipin-adipophilin-TIP47) domain, a central region of 11-mer repeats that fold into amphipathic helices (hydrophobic and hydrophilic faces on opposite sides of the helix), and a C-terminal domain that often adopts a 4-helix bundle 2, 6, 7. Historically viewed as independent modules, recent structural and functional studies indicate that these domains act cooperatively to read the local lipid environment—encompassing diacylglycerol (DAG) levels, phospholipid composition (particularly phosphatidylethanolamine (PE)), packing defects, and surface tension—for precise targeting to nascent and mature LDs 8, 9, 10, 11, 12, 13. We use the term lipid code as a convenient shorthand for this combination of compositional and biophysical cues, while recognizing that membrane binding by proteins is generally governed by such multifactorial properties.

Most membrane-associated proteins do not recognize a single lipid headgroup in isolation. They bind when several features coincide: lipid composition, packing, curvature, and electrostatics. Amphipathic helices are the most common sensors of this combination: the hydrophobic face inserts into packing defects, while the polar face remains in the aqueous phase 14, 15. On a bilayer these defects are usually small and short-lived. An LD is different. Neutral lipids in the core interdigitate with the phospholipid monolayer, surface tension rises when phospholipid coverage is limited, and packing defects become larger and more persistent 8, 12, 16. Helices that are only weakly hydrophobic, as in many PLINs, bind poorly to well-packed bilayers yet associate readily with such a tensioned monolayer. Polar residues can further stabilize the bound state through lateral contacts between neighboring helices (9). In this review, we therefore treat DAG enrichment, PE content, packing defects, and surface tension as the relevant local cues, not as a separate physical principle.

LD biogenesis begins at specialized endoplasmic reticulum (ER) subdomains where neutral lipid synthesis by ER-resident enzymes (*e.g.*, diacylglycerol acyltransferases (DGATs)) drives lens formation between the bilayer leaflets. Local lipid composition, membrane biophysical properties (surface tension and curvature), and protein machineries, including seipin complexes and Fat storage-Inducing Transmembrane (FIT) proteins, regulate subsequent budding of nascent LDs toward the cytoplasm, where they acquire their characteristic phospholipid monolayer 17, 18, 19, 20.

How a TAG lens becomes a budded droplet is still described by more than one model. In the prevailing seipin-dependent pathway, oligomeric seipin complexes mark ER subdomains, concentrate neutral lipid, and control the direction and timing of emergence; FIT proteins and Pex30 contribute to the same platforms 17, 18, 19, 20. Under high rates of TAG synthesis, droplets can also form at sites that are not stably occupied by seipin, which implies that a critical local concentration of neutral lipid can be sufficient for nucleation. These models differ in a way that matters for PLINs. If budding is seipin-templated, coat proteins may be recruited to a preexisting ER domain before a monolayer exists. If budding is driven mainly by lipid demixing, the first stable binding surface may already be an oil–water interface. Current data do not order these events: DAG-rich, seipin-positive ER patches can be detected before a droplet is optically resolved, but it remains unclear whether protein binding creates those patches, stabilizes them, or arrives after lipid demixing has begun (21).

PLINs play critical stabilizing and facilitative roles throughout this process, acting as early markers and coat proteins that read a complex lipid code. PLIN3 is rapidly recruited to sites of neutral lipid accumulation and serves as a hallmark of nascent LDs. Through its PAT domain, PLIN3 preferentially associates with DAG-enriched ER membrane regions that coincide with LD biogenesis sites containing seipin, which organizes nucleation sites and stabilizes ER–LD contacts, and Pex30; by binding DAG, it may help concentrate these factors and stabilize the specialized ER domains 21, 22. These domains have been described as exhibiting LD-like properties (local enrichment of neutral-lipid precursors, increased packing defects, and elevated surface tension) even before full budding, which may facilitate efficient neutral-lipid partitioning (21). Whether PLIN binding actively generates or merely stabilizes such domains remains an open question. Depletion of PLIN3 impairs TAG incorporation into nascent LDs, leading to fewer and smaller droplets, whereas its expression in heterologous systems is sufficient to promote LD induction and maturation 23, 24. This highlights an active, biogenesis-promoting role for PLIN3 that extends well beyond passive surface coating.

In this review, we synthesize recent advances in PLIN domain structures, lipid-binding specificities, and putative cooperative functions, with emphasis on PE recognition by the 4-helix bundle and on domain synergy during LD assembly 13, 25, 26, 27. We use the term cooperative to indicate that the domains need not act as independent, interchangeable modules whose outputs simply sum; rather, engagement of one domain can allosterically or conformationally influence the lipid-binding competence of others. The clearest illustration is the coupled disorder-to-order transition that spans the PAT domain and the 11-mer repeats of PLIN3 upon recognition of DAG-containing membranes (10). This is mechanistically distinct from a purely combinatorial code, in which each domain would contribute an independent affinity increment. Understanding these mechanisms provides fresh perspectives on LD biology and therapeutic targets for lipid storage diseases.

This review is part of the JBC Reviews Series honoring the memory of Dr Susan A. Henry, a visionary scientist and pioneer in the genetic regulation of lipid metabolism. Using yeast as a model system, Dr Henry elucidated fundamental mechanisms governing phospholipid synthesis and the cellular response to inositol, discoveries that established key frameworks for understanding lipid homeostasis in all eukaryotes, including the biogenesis and regulation of LDs (28). Her groundbreaking work, mentorship, and leadership continue to inspire research in this field.

## The perilipin family: Domain architecture and functional diversity

The PLIN family comprises five members in mammals (PLIN1–PLIN5) that share a largely common modular architecture but have diverged to fulfill specialized roles adapted to the metabolic needs of different tissues 2, 7. This domain organization and LD-targeting capacity are functionally conserved from mammals to *Drosophila* and *Dictyostelium* (29). Most PLINs feature an N-terminal PAT domain, a central region rich in 11-mer repeats that form amphipathic helices, and a C-terminal domain frequently organized as a 4-helix bundle, with notable isoform-specific modifications such as the dramatically extended repetitive region in PLIN4, hydrophobic segments in PLIN1, and a C-terminal membrane-binding domain (MBD) in PLIN5 (Fig. 1*A*) 6, 8, 30, 31. The domain organization is accompanied by distinct tissue expression profiles, highlighting the specialization of individual PLIN isoforms across tissues (Fig. 1*B*).

**Figure 1:**
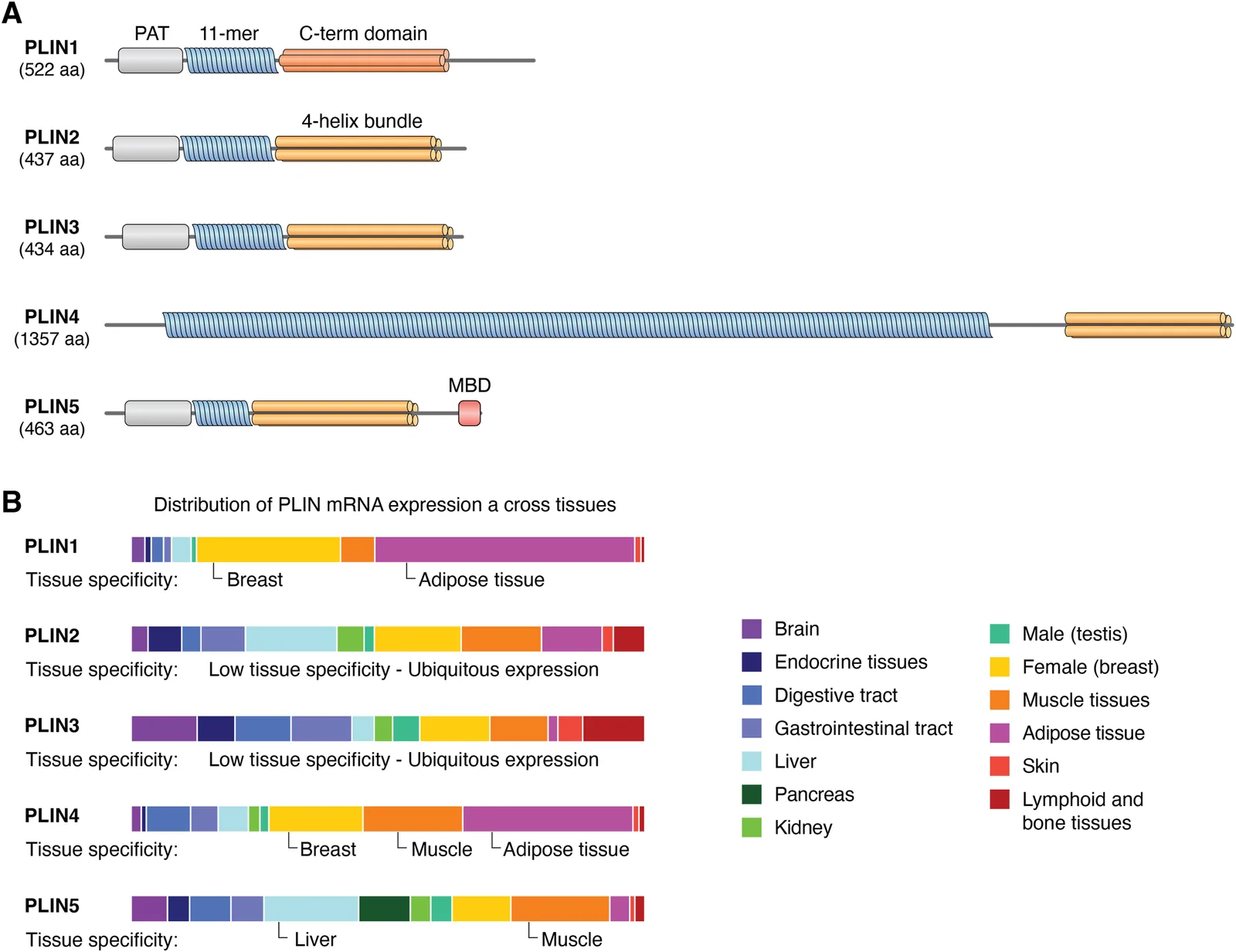
**Domain organization and tissue expression profiles of human perilipins (PLINs).** (*A*) Schematic domain architecture of PLIN1–PLIN5, shown to scale according to protein length. The conserved N-terminal PAT domain is shown in gray, the central 11-mer repeat region in blue, the C-terminal 4-helix bundle (or related C-terminal domain of PLIN1 containing hydrophobic segments) in orange, and the membrane-binding domain (MBD) of PLIN5 in magenta. Residue lengths are indicated. The scale highlights the substantially greater length of PLIN4 compared with the other PLIN members. (*B*) Relative mRNA expression profiles of *PLIN1–PLIN5* across human tissues. Expression levels are shown for each tissue, with different colors representing the major tissue groups (*e.g.*, brain, endocrine, digestive, and others). The width of each stacked bar represents the relative contribution of each tissue category to the total *PLIN* mRNA expression profile, based on normalized expression values expressed in arbitrary units. The tissue-specificity annotations summarize the predominant tissue categories evident from the expression profiles. The profiles highlight strong enrichment of *PLIN1* in adipose tissue, broader expression of *PLIN2* and *PLIN3*, tissue-enhanced expression of *PLIN4* in adipose tissue, breast, and skeletal muscle, and enrichment of *PLIN5* in oxidative tissues. Expression data were obtained from the Human Protein Atlas consensus dataset, which integrates transcriptomic data from HPA and GTEx (78).

PLIN1 (perilipin) is highly expressed in adipocytes, where it coats large, mature LDs and regulates lipolysis through interactions with adipose triglyceride lipase (ATGL) and hormone-sensitive lipase (HSL) 6, 32, 33, 34, 35. Consistent with this functional specialization, the tissue expression profile shows strong enrichment of PLIN1 in adipose tissue (Fig. 1*B*). Unlike PLIN2–5, which reach LDs from the cytosol, PLIN1 is inserted into the ER and moves onto droplets along the ER–LD continuum 31, 36. We use the conventional distinction here: Class I LD proteins (PLIN1) access the droplet surface from the ER membrane, whereas Class II LD proteins (PLIN2–5) bind the monolayer directly from the cytoplasm (37). An unconventional integral membrane segment confers Class I behavior on PLIN1, supports high-affinity coating, and produces distinct LD populations upon fatty-acid uptake while excluding other PLINs 31, 36. This ER-insertion route places PLIN1 upstream of the monolayer-based lipid-code sensing used by soluble Class II isoforms. Because PLIN1 reaches the LD surface *via* the ER continuum rather than by direct cytosolic binding, its targeting is only partly dependent on the packing-defect and surface-tension cues that dominate recruitment of PLIN2–5.

PLIN2 (adipophilin, also known as Adipose Differentiation-Related Protein, ADRP) and PLIN3 (also known as Tail-Interacting Protein of 47 kDa, TIP47) are ubiquitously expressed (Fig. 1*B*) 38, 39. The PLIN2 isoform is associated with protective functions on intermediate LDs 40, 41. Parallel CRISPR-Cas9 screens in PLIN2 reporter cells identified transcriptional and post-translational regulators of PLIN2 abundance and LD number, including the ER-associated degradation (ERAD) E3 ligase MARCH6 (42). MARCH6 limits TAG biosynthesis; when it is active there are fewer droplets and more PLIN2 turnover. It is therefore an indirect stabilizer of PLIN2, not the E3 that ubiquitinates PLIN2 itself. The same study released CRISPRlipid as an online data commons for lipid-related genetic screens (42).

PLIN3 (TIP47) marks nascent LDs and senses DAG for early recruitment (22). It is rapidly recruited to sites of neutral lipid accumulation and is widely used as a marker of early LD biogenesis.

PLIN4 (also known as S3-12) is a notable exception to this modular architecture: it lacks the N-terminal PAT domain and possesses a dramatically expanded central amphipathic helix built from 29 tandem 33-residue repeats (8). As highlighted by the scaled domain architecture in Figure 1*A*, PLIN4 is substantially longer than the other PLIN isoforms. Its Human Protein Atlas profile is tissue-enhanced in adipose tissue, breast, and skeletal muscle (Fig. 1*B*), which further distinguishes PLIN4 from the more broadly expressed PLIN2 and PLIN3. Consequently, PLIN4 primarily engages the packing-defect/tension cue and appears to have evolved compensatory features, polar-residue-mediated multimerization and extreme length, that potentially allow its expanded amphipathic helices to function effectively as a standalone coat 8, 9.

PLIN5 (also known as OXPAT or LSDP5) is enriched in oxidative tissues such as heart and skeletal muscle (Fig. 1*B*). It facilitates LD–mitochondria tethering, thereby supporting fatty acid oxidation 43, 44, 45, 46. This tethering activity is conferred by a short, PLIN5-specific C-terminal membrane-binding domain (MBD; also called the mitochondrial tether domain) that extends beyond the conserved 4-helix bundle (Fig. 1*A*). Domain-swap and truncation experiments showed that the extreme C-terminal ∼20 residues of PLIN5 are required to recruit mitochondria to LDs and that grafting this segment onto PLIN2 is sufficient to confer mitochondrial clustering (44). Subsequent mapping defined a minimal tether that binds the mitochondrial acyl-CoA synthetase FATP4 (ACSVL4); the two C-terminal domains constitute a minimal pair capable of inducing organelle contacts (30). The MBD is therefore not a primary reader of the DAG, packing-defect, and PE cues that recruit PLIN5 to the LD monolayer. It is an isoform-specific extension that couples an already LD-bound coat to mitochondria, allowing phosphorylation-gated fatty-acid channeling during starvation or increased energy demand 30, 44.

These structural variations, together with distinct tissue expression patterns, summarized in Figure 1 and Table 1, underpin functional diversity and hierarchical LD targeting (47). Recent studies emphasize that the domains act in concert rather than as independent modules, allowing PLINs to integrate lipid cues such as DAG enrichment, packing defects, surface tension, and PE composition 12, 13. This specialization, refined in vertebrates, supports complex lipid metabolism and LD heterogeneity 48, 49. Understanding these domain features provides a framework for dissecting how PLINs sense and respond to the local lipid environment during LD assembly.

**Table 1:** Domain architecture, tissue expression, lipid-sensing properties, and functional specialization of human perilipin isoforms

Isoform	Domain architecture	Primary tissue expression	Preferred LD stage/main role	Key lipid cues sensed	Distinctive features	Key references
PLIN1	PAT 11-mer AHs C-term iMS	Adipocytes (high)	Mature, large LDs; regulates lipolysis (ATGL/HSL)	High-affinity monolayer binding; less sensitive to tension than Class II PLINs	Class I protein; ER insertion *via* iMS ER-to-LD pathway; creates distinct LD populations and excludes other PLINs	31, 32, 34
PLIN2	PAT 11-mer AHs 4HB	Ubiquitous	Intermediate LDs; protective/stability functions	Packing defects, surface tension, PE (*via* 4HB)	Regulated by ERAD (*e.g.*, MARCH6); intermediate affinity	(38, 40, 41, 42)
PLIN3	PAT 11-mer AHs 4HB	Ubiquitous	Nascent/early LDs; promotes biogenesis platforms	DAG (PAT), packing defects/tension (AHs), PE (4HB cleft)	Rapid recruitment to DAG-rich ER subdomains; cooperates with seipin/Pex30; best-characterized cooperative sensor	10, 11, 13, 21, 22, 62
PLIN4	No PAT Expanded 11-mer 4HB	Tissue-enhanced in adipose tissue, breast, and skeletal muscle	Nascent or rapidly expanding/phospholipid-limited LDs	Packing defects and high surface tension	Extreme length and polar-residue-mediated multimerization; can partially substitute for limiting phospholipid monolayer; exceptional surface stability	8, 9, 12, 62
PLIN5	PAT 11-mer AHs 4HB MBD	Oxidative tissues (heart, skeletal muscle)	LDs in contact with mitochondria; facilitates fatty-acid oxidation	Packing defects/tension, PE (inferred)	C-terminal MBD tethers LDs to mitochondria, in part *via* FATP4; tissue-specialized metabolic role	30, 43, 44, 46

4HB, 4-helix bundle; AH, amphipathic helix; ADRP, adipose differentiation-related protein; ATGL, adipose triglyceride lipase; DAG, diacylglycerol; ERAD, endoplasmic reticulum-associated degradation; HSL, hormone-sensitive lipase; iMS, integral membrane segment; LD, lipid droplet; MBD, membrane-binding domain; PAT, Perilipin–ADRP–TIP47 domain; PE, phosphatidylethanolamine; TIP47, tail-interacting protein of 47 kDa.

## The PAT domain: Sensing diacylglycerol for nascent LD targeting

The N-terminal PAT domain is a defining feature of most PLINs and plays a pivotal role in the initial targeting to sites of LD biogenesis at the ER 2, 6, 50. Structural predictions and biochemical studies indicate that the PAT domain adopts a compact, triangular amphipathic fold that preferentially interacts with DAG, a key intermediate in TAG synthesis (Fig. 2*A*) 10, 11. The hydrophobic and hydrophilic residues are preferentially distributed on opposite faces of the PAT domain, providing a structural feature that may facilitate membrane association by allowing the hydrophobic surface to engage the lipid phase while the hydrophilic surface remains exposed to the aqueous environment (Fig. 2*A*).

**Figure 2:**
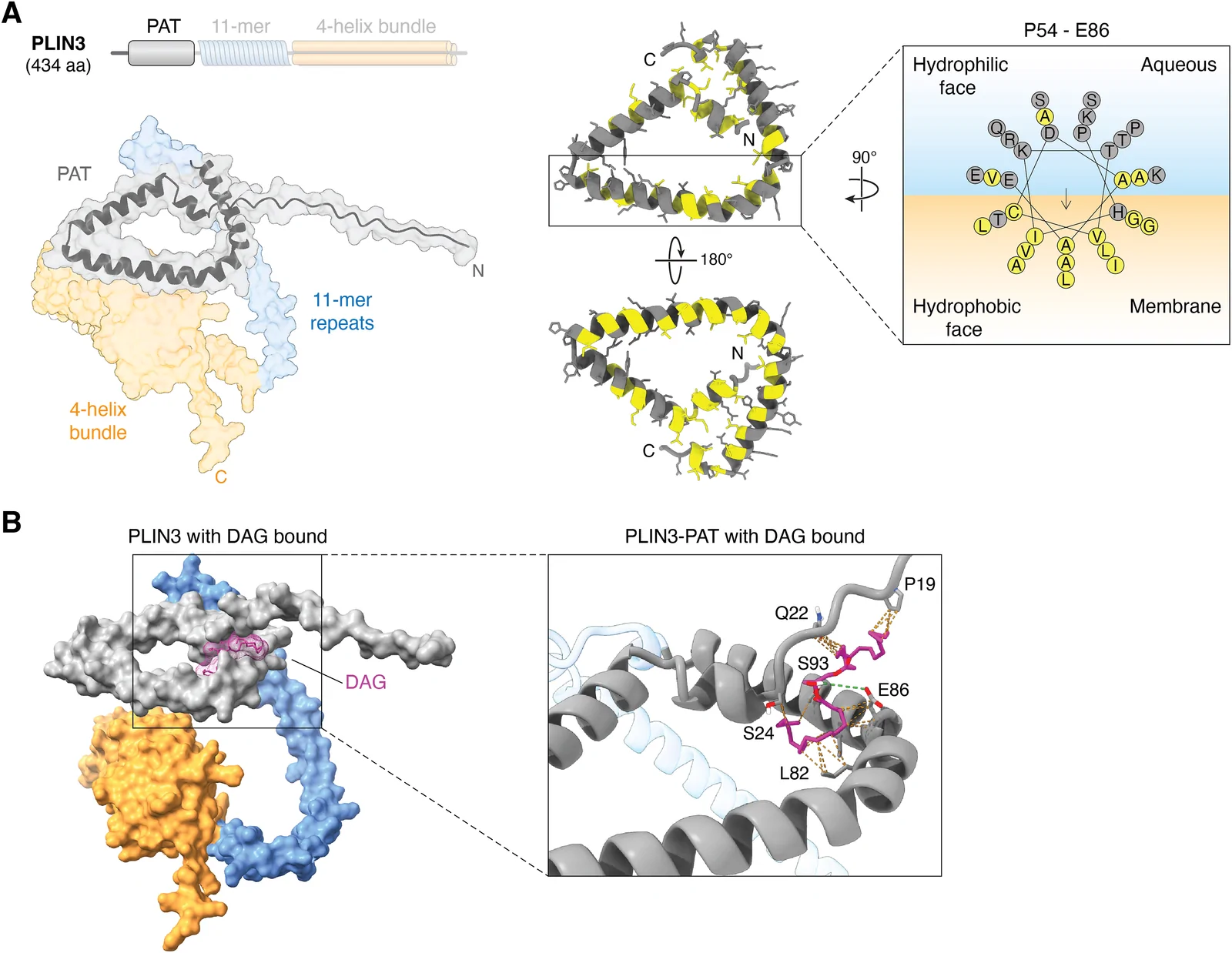
**Amphipathic character and membrane-interaction features of the PAT domain.***A*, schematic representation of the domain architecture of PLIN3, with the PAT domain (perilipin-adipophilin-TIP47) highlighted in *gray* and the 11-mer repeats and 4-helix bundle shaded. The corresponding structural model of PLIN3 highlights the PAT domain (*left panel*), which is further shown in two orientations related by a 180° rotation (*middle panel*). The PAT domain displays an amphipathic character, with hydrophilic (in *gray*) and hydrophobic residues (in *yellow*) preferentially distributed on opposite faces of the structure. The corresponding helical-wheel projection of the amphipathic segment, generated using HeliQuest (79), illustrates the segregation of hydrophobic and polar/charged residues (*right panel*). Such spatial segregation of polar and nonpolar residues can facilitate association with membrane surfaces by allowing the hydrophobic face to interact with the lipid phase while the hydrophilic face remains exposed to the aqueous environment. *B*, structural model of full-length PLIN3 with an enlarged view of the PAT domain illustrating complementary features of its lipid and membrane interactions. The PAT domain is shown in complex with diacylglycerol (DAG; magenta), with key lipid-contacting residues labeled. Adapted from (11).

In PLIN3, the PAT domain preferentially engages DAG-enriched ER subdomains, facilitating recruitment prior to or during neutral lipid lens formation (22). Structural modeling further identifies a DAG-binding site within the PAT domain, with specific residues contacting DAG (Fig. 2*B*) (11). This DAG-dependent recruitment may contribute to the assembly of LD biogenesis platforms that include seipin, Pex30, and other factors, driving the transition from DAG-rich membrane domains to nascent LDs 17, 20, 51. Recent work in yeast and mammalian systems shows that membrane-anchored PLIN3 induces expansion and vesiculation of perinuclear ER membranes into crescent-shaped domains enriched in DAG and LD marker proteins, which readily convert into LDs upon TAG synthesis induction 10, 21.

Selectivity for DAG over other phospholipids is conferred by conserved acidic residues (Glu/Asp) that coordinate the DAG headgroup within a curved surface of the PAT domain (11). Point mutations of these residues impair DAG binding and reduce PLIN3 association with DAG-containing membranes and artificial LDs, consistent with a role in sensing DAG-enriched ER subdomains during early LD biogenesis (11). In addition to this defined lipid-binding site, protein–membrane interaction prediction identifies distinct regions within the PAT domain with different predicted membrane-interaction behaviors (52). These predictions suggest that the PAT domain may contain multiple membrane-interacting elements that extend beyond direct DAG recognition and could contribute to membrane engagement and potentially facilitate responses to local membrane properties. Thus, DAG recognition and broader membrane-interaction features may act together to promote PLIN3 association with the ER and nascent LD surface. These properties may help explain why PLIN3 is a reliable marker of early LD formation (22).

Current evidence supports a model in which the PAT domain acts as an initiating module: its engagement with DAG-rich membranes can promote a conformational change that facilitates subsequent insertion of the 11-mer repeats. However, alternative models in which the domains bind more independently remain possible. Isoform-specific differences in PAT domain affinity might further contribute to the functional specialization observed across the PLIN family 2, 10, 11.

## The 11-mer repeat region: Amphipathic helices and recognition of lipid packing defects

The central region of PLINs contains multiple 11-mer repeats that fold into amphipathic helices upon membrane interaction 8, 53. Early domain-mapping studies on PLIN2 already identified central α-helical segments as critical for LD targeting (54). These amphipathic helices constitute the primary interface with the LD phospholipid monolayer and are critical for sensing the unique biophysical properties of the LD surface, particularly surface tension and lipid packing defects (Fig. 3) 12, 14, 15. As for the PAT domain, the amphipathic helices formed by the 11-mer regions contain hydrophobic and hydrophilic residues preferentially distributed on opposite faces, allowing the hydrophobic face to interact with the membrane lipids while the hydrophilic face remains exposed to the aqueous environment (Fig. 3*A*). The physical properties of the LD surface provide an important context for this mode of membrane association. Unlike a phospholipid bilayer, an LD consists of a phospholipid monolayer surrounding a neutral-lipid-rich core, with interdigitation between surface phospholipids and underlying neutral lipids contributing to its distinctive surface properties (16). Increased surface tension can reduce phospholipid packing and create hydrophobic voids within the monolayer, while promoting interdigitation of core neutral lipids into these regions (Fig. 3*B*) (16). These changes in lipid packing may increase the accessibility of neutral lipids at the interface and provide favorable hydrophobic environments for association of amphipathic helices with the LD surface. Thus, the transition of an 11-mer region from an unfolded or soluble state to an amphipathic α-helical conformation provides a potential mechanism for coupling PLIN binding to the physical state of the LD surface (Fig. 3*B*) 8, 9, 14.

**Figure 3:**
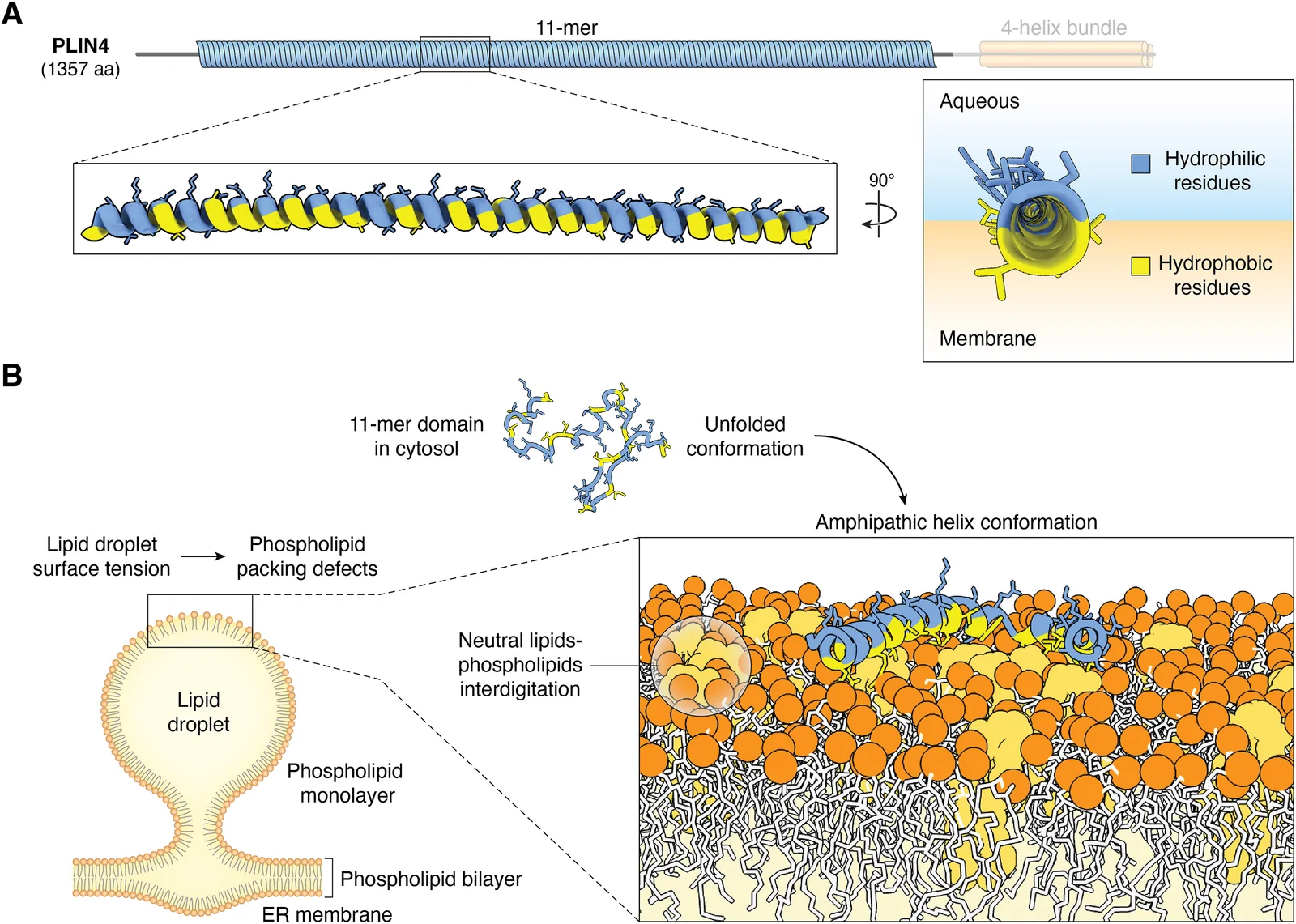
**Amphipathic helices formed by the 11-mer repeat regions and their engagement with the lipid-droplet surface.***A*, schematic representation of the PLIN4 domain architecture, shown to scale, with the expanded 11-mer repeat region highlighted. An enlarged view of a representative segment of the 11-mer repeat region illustrates its amphipathic character, with hydrophobic (in *yellow*) and hydrophilic residues (in *blue*) preferentially distributed on opposite sides of the helix. The amphipathic α-helix can therefore present a hydrophobic face toward the lipid phase while retaining a hydrophilic face exposed to the aqueous environment. *B*, conceptual model of the conformational transition of an 11-mer region from an unfolded soluble state to an amphipathic α-helical conformation upon contact with the lipid-droplet (LD) surface. The model illustrates how increased LD surface tension reduces phospholipid (PL) packing, creating hydrophobic voids within the monolayer and promoting interdigitation of core neutral lipids (NL) into these regions. These tension-dependent packing defects and the increased exposure of NL at the interface may provide favorable hydrophobic environments for association of amphipathic helices with the LD surface. This conceptual framework is supported by biophysical and simulation studies of PLIN amphipathic helices and LD surface properties 8, 9, 12, 14, 16.

Unlike classical amphipathic helices in proteins that target bilayer membranes, PLIN amphipathic helices are often weakly hydrophobic, particularly in PLIN4, which features an exceptionally long and repetitive amphipathic helices (29 tandem 33-mer repeats) lacking bulky hydrophobic residues (Fig. 3*A*) (8). This adaptation may favor association with LDs, which exhibit higher surface tension and more packing defects than ER or other organelle bilayers, especially during growth phases when phospholipid coverage becomes limiting 12, 55. *In vitro* studies with synthetic lipids demonstrate that purified PLIN4 amphipathic helices bind poorly to phospholipid bilayers but strongly interacts with pure triglycerides, effectively acting as a coat and stabilizing small oil droplets (8). Consistent with this behavior, PLIN4 overexpression can alleviate LD instability under conditions of limited phospholipid coverage, suggesting that its expanded amphipathic helices can partially compensate for reduced phospholipid surface coverage 8, 55.

Importantly, the sensitivity to surface tension is not merely quantitative. Purified PLIN3 and PLIN4 amphipathic helices bind far more avidly to tensioned, sparsely phospholipid-covered droplets, whereas PLIN1 associates more readily with droplets whose monolayer is already fully saturated (12). This provides a biophysical explanation for the observed cellular distribution in which PLIN3 and PLIN4 preferentially mark nascent or rapidly expanding LDs, while PLIN1 is enriched on large, mature droplets 12, 48.

Polar residues within the amphipathic helices play an unexpectedly important role in stability. In PLIN4, the precise distribution and nature of these polar residues are proposed to enable multimeric assembly of adjacent amphipathic helices through polar/electrostatic interactions, reminiscent of apolipoproteins in lipoprotein particles (9). This lateral cooperation results in exceptional immobility and stability of PLIN4 on LD surfaces, as revealed by fluorescence-recovery-after-photobleaching and *in vitro* reconstitution experiments (9). Collectively, current evidence supports the view that the 11-mer amphipathic helices behave as dynamic sensors of lipid packing defects and surface tension, contributing to isoform-specific targeting and adaptation. Alternative or additional mechanisms, including protein–protein interactions and post-translational modifications, are also likely to influence binding specificity 12, 56. PLIN3 and PLIN4, with their more flexible amphipathic helices, preferentially associate with nascent or expanding LDs, while other isoforms display distinct affinities. This domain works cooperatively with the PAT domain (for initial DAG sensing) and the C-terminal 4-helix bundle (for stable PE-sensitive anchoring), enabling PLINs to respond to the evolving biophysical properties of the LD surface during biogenesis, growth, and maturation. These properties help explain the preferential distribution of PLIN isoforms across heterogeneous LD populations 12, 47, 48.

## The C-terminal 4-helix bundle: Phosphatidylethanolamine binding and stable LD anchoring

The C-terminal domain of most PLINs adopts a structured 4-helix bundle that has apolipoprotein-like properties and provides stable anchoring to the LD surface 23, 25. The crystal structure of the PLIN3/TIP47 C-terminal domain showed that this bundle is structurally related to the N-terminal 4-helix bundle of Apolipoprotein E (ApoE) and to insect apolipophorin III, despite little sequence identity 25, 57, 58. In lipid-free ApoE the four amphipathic helices pack against one another and bury a hydrophobic core; lipid association opens the bundle so that helix–helix contacts are replaced by helix–lipid contacts (58). That reversible switch is the precedent for treating the PLIN 4-helix bundle as a lipid-binding motif rather than only a protein–protein scaffold 26, 58, 59. Two differences should be kept in view. First, the ApoE bundle is N-terminal and works together with a separate C-terminal amphipathic domain that initiates lipoprotein binding, whereas the PLIN bundle is C-terminal and is likely recruited after the PAT domain and 11-mer repeats have engaged the monolayer. Second, whether the PLIN bundle undergoes a comparable wholesale opening on LDs, or instead uses the conserved hydrophobic cleft to bind PE without unfolding, has not been resolved structurally 13, 25.

A conserved hydrophobic cleft within the 4-helix bundle was first visualized in the PLIN3/TIP47 crystal structure (25) and later highlighted as a putative lipid-binding site by Ohsaki *et al.* (2006) (26). Building on that structural work, pendant-drop tensiometry showed that the isolated PLIN3 4-helix bundle prefers PE-containing interfaces (27). AlphaFold modeling, docking, and mutagenesis place that preference on the hydrophobic cleft first seen in the crystal structure (Fig. 4*A*) 13, 25, 26. The PE bias is partly masked in the full-length protein, which indicates that the PAT domain and 11-mer repeats influence the lipid specificity of the bundle rather than acting as fully independent modules (13).

**Figure 4:**
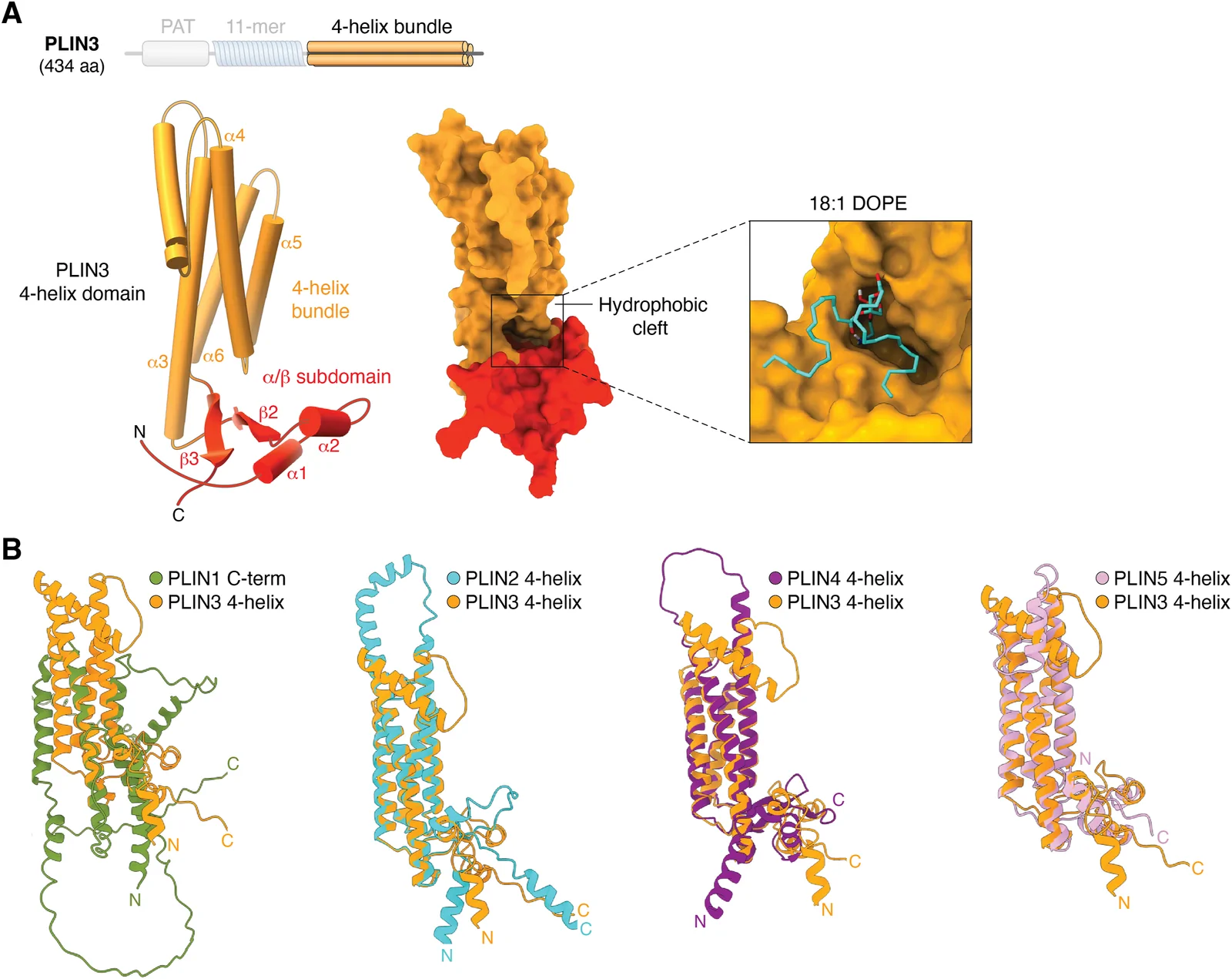
**Structure of the PLIN 4-helix bundle domain and recognition of phosphatidylethanolamine (PE) in a hydrophobic cleft.***A*, schematic representation of the PLIN3 domain architecture, with the 4-helix bundle domain highlighted, followed by a ribbon representation of the PLIN3 4-helix bundle (α3–α6, *orange*) and adjacent α/β subdomain (*red*). Secondary-structure elements are simplified, with α-helices shown as cylinders and β-sheets as arrows. Surface rendering highlights the hydrophobic cleft. Close-up views show the cleft occupied by 18:1 DOPE (1,2-dioleoyl-sn-glycero-3-phosphoethanolamine) (*cyan*), as identified by *in silico* docking simulations. The panel is adapted from (13). *B*, superposition of the AlphaFold predicted structures of C-terminal domains of PLIN family members onto the PLIN3 4-helix bundle scaffold, illustrating conservation of the overall bundle architecture among PLIN2, PLIN3, PLIN4, and PLIN5, and the distinct fold of PLIN1. The panel is adapted from (13).

The cleft therefore remains a candidate PE-binding site, but the cellular evidence is still indirect. Mutagenesis of cleft-lining residues impairs LD targeting, and lowering PE reduces PLIN recruitment (13). That result is consistent with earlier measurements showing that a decreased phosphatidylcholine-to-PE ratio at the LD surface correlates with increased PLIN2 recruitment (60). *In vitro*, purified 4-helix bundles bind PE-enriched artificial LDs more readily than droplets made with other phospholipids 13, 27. These findings support a role for the 4-helix bundle as the module conferring long-term stability after initial recruitment by the PAT domain and 11-mer amphipathic helices (13).

Isoform-specific adaptations could modulate the function of this C-terminal 4-helix bundle. Structural comparison shows conservation of the overall 4-helix bundle architecture and of the residues lining the hydrophobic cleft among PLIN2, PLIN3, PLIN4, and PLIN5, whereas PLIN1 adopts a distinct C-terminal fold (Fig. 4*B*) (13). PLIN1 contains additional C-terminal hydrophobic segments that promote integral membrane behavior and high-affinity LD binding, contributing to the formation of distinct LD populations in adipocytes 31, 36, 48. In PLIN3, the bundle works synergistically with the PAT domain and amphipathic helices to achieve robust localization. The PE-binding cleft likely stabilizes the protein on the curved, tensioned LD monolayer and may facilitate lateral protein-protein interactions that further reinforce the coat 58, 61.

Recent comparative analysis of the 4-helix bundles of PLIN3 and PLIN4 further refined this picture (62). The PLIN3 4-helix bundle is a stably folded domain that binds lipid surfaces *in vitro* and localizes to LDs in cells. An αβ-subdomain at the base of the bundle is required for LD binding but is dispensable for overall fold stability, suggesting that this region contributes directly to membrane engagement. In contrast, the corresponding region in PLIN4 is predicted to be less structured and less stable during MD simulations and does not support LD binding under the conditions tested (62). These findings reinforce the view that PLIN4 depends primarily on its expanded amphipathic helix for surface association, while the 4-helix bundle plays a more prominent anchoring role in PLIN3 and related isoforms.

Collectively, the 4-helix bundle completes a multivalent lipid-sensing mechanism. By integrating PE recognition with upstream DAG sensing (PAT) and packing-defect detection by amphipathic helices, PLINs achieve precise, hierarchical targeting and stable association with LDs of varying composition and maturation states. Current evidence supports the idea that this domain synergy contributes to LD assembly, heterogeneity, and metabolic regulation, although additional regulatory layers and alternative targeting mechanisms are also likely to operate.

## Cooperative domain functions in reading the lipid code for LD assembly

The three principal domains of PLINs appear to function as an integrated sensor system that interprets the evolving lipid environment at the ER and LD surface to orchestrate precise targeting, LD budding, growth, and maturation (Fig. 5). This synergy enables PLINs to respond dynamically to local changes in DAG levels, phospholipid composition (particularly PE), lipid packing defects, and surface tension.

**Figure 5:**
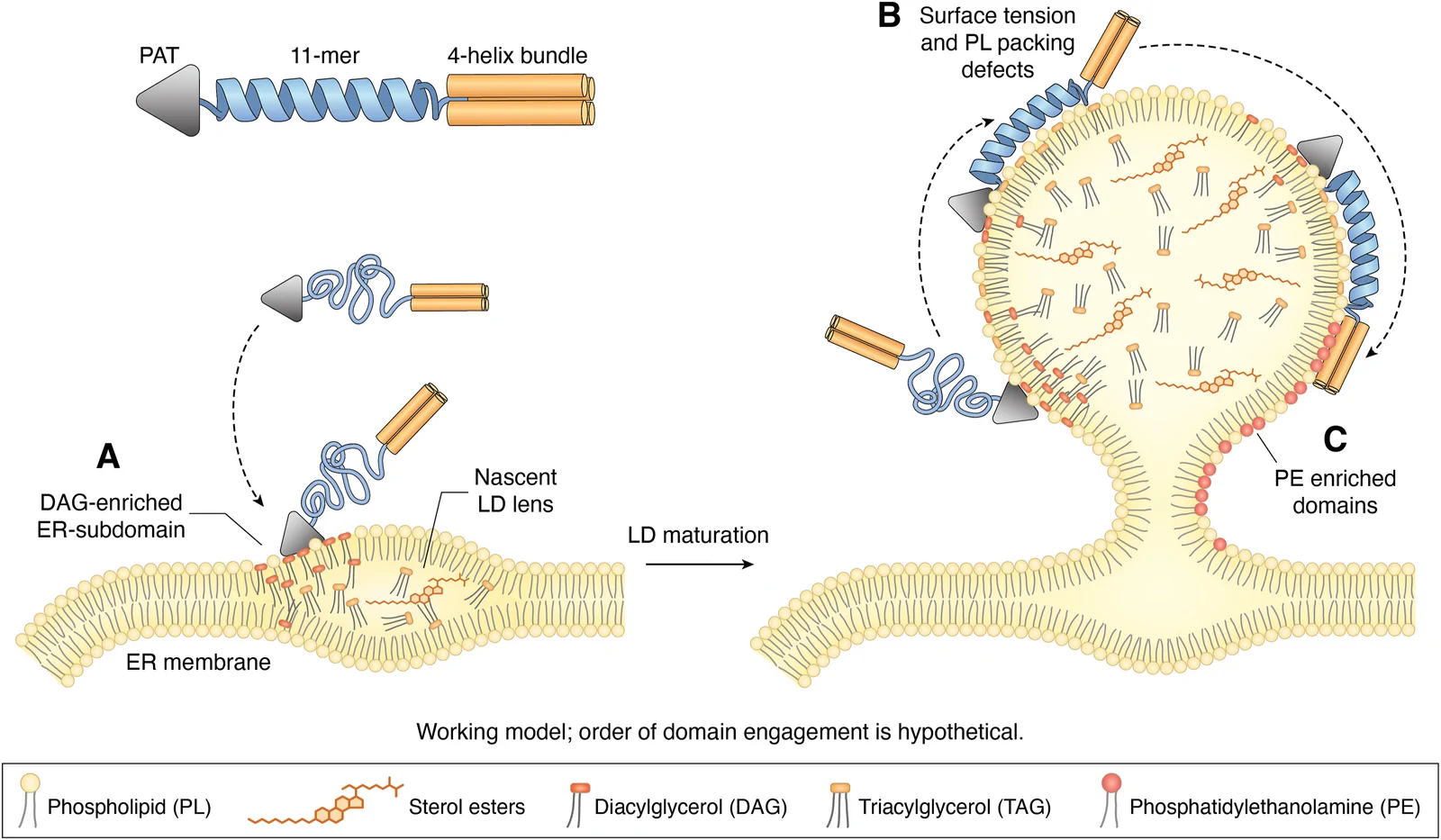
**Cooperative domain functions during lipid-droplet biogenesis and maturation.** Schematic model illustrating how the three major domains of PLINs cooperatively read the evolving lipid code as lipid droplets (LDs) form and mature. *Top left*: Domain architecture of a typical PLIN (PAT domain, 11-mer repeats, 4-helix bundle). *A*, *DAG enrichment* (*left*): Local accumulation of diacylglycerol (DAG) in the endoplasmic reticulum bilayer recruits the PLIN, primarily *via* the PAT (Perilipin–ADRP–TIP47) domain. The 11-mer and 4-helix domains are shown beginning to engage the nascent neutral-lipid lens. *LD maturation* (*center arrow*): Transition from a nascent LD lens within the bilayer to a mature, budded LD. *B*, *Surface tension & lipid packing defects* (*upper right*): On the mature LD the phospholipid monolayer experiences elevated surface tension and packing defects. These features are sensed and stabilized by the amphipathic 11-mer repeats (*blue*). *C*, *PE*-*enriched domains* (*right*): Clustering of phosphatidylethanolamine (PE, *red*) creates preferred binding sites that are recognized by the hydrophobic cleft of the 4-helix bundle (*orange*), further anchoring the protein. Dashed lines indicate the transition of individual domains from non-membrane associated to membrane-associated during LD binding of PLINs. Lipid symbols are defined in the key below the figure (DAG, diacylglycerol; PE, phosphatidylethanolamine; PLs, phospholipids; STE, sterol esters; TAG, triacylglycerol). This schematic is a working model, not a demonstrated kinetic sequence. It shows one plausible order in which the PAT domain, 11-mer repeats, and 4-helix bundle could engage DAG, packing defects/surface tension, and PE, respectively. The strongest current evidence for true cooperativity, rather than additive independent binding, is the coupled disorder-to-order transition spanning the PAT domain and 11-mer repeats of PLIN3 on DAG-containing membranes (10). Independent or partially overlapping domain engagement has not been excluded. The framework integrates structural and biophysical data on domain-specific lipid recognition 8, 11, 13, 47.

Initial recruitment is driven by the PAT domain, which senses DAG enrichment in specialized ER subdomains that overlap with biogenesis platforms containing seipin and other factors 10, 11, 21, 22. Once positioned near the nascent neutral lipid lens, the 11-mer amphipathic helices insert into regions of high surface tension and packing defects that characterize early LD surfaces (12). In isoforms such as PLIN4, the extended repetitive amphipathic helices further enable multimeric lateral interactions *via* polar residues, conferring exceptional stability and the ability to partially substitute for limiting phospholipids during rapid LD expansion 8, 9.

Stable anchoring is then reinforced by the C-terminal 4-helix bundle, whose hydrophobic cleft can engage PE, a cone-shaped lipid enriched at curved, PE-rich interfaces 13, 27, 60. This sequential and synergistic engagement provides a mechanistic framework for the preferential targeting of PLIN isoforms, PLIN3/4 on nascent or high-tension LDs *versus* PLIN1 on mature, fully coated LDs, as detailed above and driven in part by PLIN1’s integral membrane segment 12, 31. Consistent with non-additive domain function, the PE preference of the isolated 4-helix bundle is partly masked in the full-length protein (13).

This integrated lipid-code reading mechanism helps explain how PLINs actively participate in LD assembly while adapting to the biophysical properties of heterogeneous LD populations, providing a unified framework supported by structural modeling, *in vitro* reconstitution, and cellular mutagenesis studies. However, these data do not exclude alternative models in which domain cooperation is partially overlapping or context-dependent rather than strictly sequential. A direct test of ordered engagement would be whether 11-mer insertion still occurs when PAT–DAG recognition is blocked; persistent binding under that condition would favor independent rather than sequential domain use.

## Implications for metabolic diseases and therapeutic targeting

Dysregulation of PLIN function and LD homeostasis is strongly implicated in prevalent metabolic disorders, including MASLD, obesity, lipodystrophies, type 2 diabetes, and cancer 63, 64, 65. The cooperative lipid-code framework is useful here only where a disease phenotype can be mapped, even provisionally, onto a defined domain–lipid interaction 2, 10, 11, 12, 13.

For PLIN1 the mapping is comparatively direct. Loss- or gain-of-function variants cause familial partial lipodystrophy, with severe dyslipidemia and early cardiovascular disease 66, 67, 68. The Class I integral membrane segment and the resulting high-affinity coat are required to segregate LD classes and to restrain lipolysis in adipocytes; disrupting those features collapses that hierarchy (31). How individual patient variants act on the integral membrane segment *versus* later lipolytic scaffolds is not fully resolved.

For PLIN2 and PLIN3, the link is still largely correlative. Both accumulate on hepatic LDs as MASLD progresses 63, 65, 69. A plausible, but untested, reading is that the elevated DAG and altered PE/PC ratios of steatotic ER shift PAT-domain occupancy (PLIN3) and 4-helix-bundle anchoring (PLIN2/3), thereby stabilizing an enlarged hepatic LD pool. PLIN2 abundance is also set indirectly by TAG synthesis through the ERAD ligase MARCH6 (42), so changes in neutral-lipid flux can alter coat composition without any mutation in PLIN2 itself.

PLIN4 offers a coat-coverage mapping rather than a lipid-ligand mapping. A 3′-UTR variant that lowers PLIN4 expression through a microRNA-522 site is associated with obesity-related phenotypes (70). Given that the expanded 11-mer helix can stabilize droplets when phospholipid coverage is limiting 8, 9, reduced PLIN4 levels could become limited during rapid LD expansion. That causal step has not been shown in human adipose tissue.

PLIN5 is specialized for LD–mitochondria contacts in oxidative tissues 43, 44, 46, 71. LD recruitment of PLIN5 can still use the PAT, 11-mer, and PE-cleft cues defined for other Class II isoforms, whereas tethering itself is executed by the C-terminal MBD rather than by those lipid-code modules 30, 44. How disease phenotypes map onto the MBD *versus* the lipid-code domains has not been established and should not be forced onto the lipid-code model until those requirements are tested.

In cancer, PLIN2 and PLIN3 are frequently upregulated in neoplastic steatogenesis and can protect proliferating cells from lipotoxicity 72, 73, 74, 75. It is reasonable to ask whether tumor-driven changes in DAG, PE, or surface tension co-opt the same domain sensors, but this remains an extrapolation from the targeting data.

These mappings suggest where pharmacology might act: the PE-binding cleft, PAT–DAG engagement, or 11-mer multimerization. Yet no domain-selective ligand has been reported. Existing drugs such as fibrates can change PLIN3 levels indirectly (76). Any future compound would need isoform selectivity, because the same cues are used in adipose, liver, muscle, and tumors (65).

## Conclusion

The PLINs have emerged as sophisticated interpreters of the lipid code on LDs. Through the coordinated action of their three principal domains: the PAT domain sensing DAG for initial recruitment, the 11-mer amphipathic helices detecting packing defects and surface tension, and the C-terminal 4-helix bundle providing PE-sensitive stable anchoring, PLINs achieve precise, hierarchical targeting and active participation in LD biogenesis, growth, and functional specialization. This integrated mechanism helps account for isoform-specific behaviors and LD heterogeneity, as well as the remarkable stability of proteins such as PLIN4 on expanding or phospholipid-limited LD surfaces 9, 12, 31.

These advances, supported by AlphaFold modeling, *in vitro* reconstitution with artificial LDs, liposome-binding studies, and mutagenesis of key interacting residues, move the field beyond viewing PLINs as simple structural coats toward a picture of multivalent regulators that interpret local biophysical cues to orchestrate LD assembly and metabolism 10, 11, 12, 13. The cooperative lipid-code model is currently best supported for PLIN3 and PLIN4 and remains more inferential for PLIN2 and PLIN5. Decisive tests will require high-resolution structures of full-length PLINs on native monolayer surfaces, quantitative measurements of each domain’s contribution to targeting and retention *in vivo*, and live-cell time-resolved imaging that can distinguish sequential from partially overlapping domain engagement 2, 46, 77.

## Future perspectives

Beyond isolated domains and artificial droplets, the priority is to place full-length PLINs on native monolayer surfaces and to test whether domain engagement is ordered. In particular, it will be important to ask whether 11-mer insertion can still occur when PAT–DAG recognition is blocked, which would favor independent rather than sequential binding.

From a mechanistic standpoint, the next measurements should be quantitative: the contribution of each domain to targeting and retention *in vivo*, and how tension, packing defects, and PE content change those contributions across LD populations. In particular, native-membrane structural work, systematic mutagenesis, and live-cell time-resolved imaging that follow PLIN recruitment from DAG-enriched ER platforms through nascent LDs to mature droplets will be critical for testing whether domain cooperation is strictly ordered or partially overlapping.

On the translational side, quantitative mapping of how disease-associated variants alter specific domain–lipid interactions, combined with the development of isoform-selective modulators targeting the PE-binding cleft or amphipathic helices multimerization interfaces, may offer opportunities for therapeutic intervention in MASLD, lipodystrophies, and cancer. Achieving sufficient isoform selectivity and avoiding off-target effects will be critical, but such strategies could ultimately allow fine-tuning of LD dynamics and PLIN function in a tissue-specific manner. Ultimately, understanding how PLINs interpret local lipid cues will illuminate general principles of organelle biogenesis and offer new avenues to modulate lipid storage and utilization in human disease.

## Data availability

All data presented are contained within the manuscript.

## Declaration of generative AI and AI-assisted technologies in the writing process

During the preparation of this work the authors used AI-assisted technologies to improve the readability and language of the manuscript. After using this tool, the authors reviewed and edited the content as needed and take full responsibility for the content of the published article.

## Conflict of interest

The authors declare that they have no conflicts of interest with the contents of this article.
